# Prevalence and management practice of first generation antipsychotics induced side effects among schizophrenic patients at Amanuel Mental Specialized Hospital, central Ethiopia: cross-sectional study

**DOI:** 10.1186/s12888-018-1999-x

**Published:** 2019-01-18

**Authors:** Yirgalem Shewakena Wubeshet, Oumer Sada Mohammed, Tigestu Alemu Desse

**Affiliations:** Department of Clinical Pharmacy and Pharmacology, School of Pharmacy, College of Health Sciences, Addis Ababa University, Addis Ababa, Ethiopia

**Keywords:** First generation antipsychotics, Side effects, Schizophrenia, Ethiopia

## Abstract

**Background:**

First-generation antipsychotics (FGAs) are associated with a range of adverse events which can significantly reduce patients’ quality of life and contribute to non-adherence. The aim of this study was to assess the prevalence and management practice of first generation antipsychotics induced side effects among schizophrenic patients.

**Methods:**

The study was conducted at Amanuel Mental Specialized Hospital from March to June, 2017. Data from patients were collected using a pretested structured questionnaire. Demographics and side effects of antipsychotics were collected by face to face interview. Clinical characteristics, medications and previous history of adverse drug events were extracted from medical records using data abstraction format. The data were analyzed using statistical software for social sciences (SPSS) version 20. Descriptive statistics and chi-square tests were done. Statistical significance was considered at *p* < 0.05.

**Results:**

Out of 356 participants, 300 of them had complete data and were included in the study. The mean age of participants was 33.71 ± 10.2 years. The majority, 195(65.0%), of participants were males. Most of the participants, 293(97.7%), developed FGA medication induced side effects. One hundred sixty three (54.3%) participants were treated with Trihexyphenidyl for FGAs induced side effects. Dose reduction of antipsychotics was done for 51(17.0%) participants. Most of the participants’ side effects were not managed according to American Psychiatric Association guideline; 178 (82.4%). The most common types of FGAs induced side effects were cardiovascular side effects 169(56.3%); sedation and CNS side effects 149(49.6%); and extrapyramidal side effects 114(38.0%). There is a significant association between occurrence of side effects of FGAs and duration of illness (*P* = 0.04).

**Conclusions:**

The prevalence of first generation antipsychotics induced side effects was high. However, management practice of the side effects was minimal.

## Background

Antipsychotic medications have been the mainstay of treatment for schizophrenia since the introduction of chlorpromazine in the 1950s [1]. These medications still are the treatment of choice for such disorder. They reduce psychotic symptoms; improve patient’s functionality and quality of life. However, they are often associated with some intolerable side effects [2]. Such side effects include severe sedation, weight gain, sleep disorders, sexual dysfunction and difficulties in social activities [3]. The intolerability of these side effects has been identified as determinant of non-adherence to antipsychotic medication [4, 5]. Discontinuing antipsychotic medication often leads to relapse in psychotic disorders which in turn may result in loss of job, difficulty in social relationship and increased risk of suicide [3]. Schizophrenic patients are often experience illness occurrence, unendurable side effects from prescribed medication, discontinuation of medication and disease relapse [6].

Antipsychotic medications can produce a range of adverse effects that significantly affect the patient’s quality of life. They may also cause greater levels of distress than the symptoms of the illness [3, 4, 7]. Studies have shown that between 50 and 70% of schizophrenic patients experience at least one serious side-effect from antipsychotic medication [8]. Of these serious effects, the annual incidence ranges from 37 to 44% for Parkinsonism, 26 to 35% for akathisia, and 8 to 10% for tardive dyskinesia [6]. Schizophrenic patients also commonly experience other side-effects, such as weight gain, excessive sleep, insomnia, sexual dysfunction, dry mouth, constipation, urinary problems, and dizziness [9].

First-generation antipsychotic drugs are more likely to cause adverse effects such as extrapyramidal symptoms and tardive dyskinesia. The most serious side effects of FGAs are neurological and are largely confined to the extrapyramidal motor system. Although the FGAs have gradually been replaced by the second-generation (SGAs) in developed nations, they remain the most commonly prescribed medications in many parts of the world especially developing countries as they are considerably less expensive than newer antipsychotics [10–12].

Side effects of FGA medications are highly prevalent and significantly associated with lower adherence, which in turn result in increased use of healthcare resources. Prevention and monitoring, and reduction of medication side effects may lead to better adherence and improved outcomes of schizophrenic patients [13, 14]. Assessment and management of antipsychotic medication side-effects are considered essential to prevent negative physical health outcomes, improve tolerability, and promote medication adherence [6, 15]. However, previous work suggests that clinician knowledge and skill in the management of antipsychotic medication side-effects remain poorly developed [2].

While antipsychotics have similar efficacy, adverse effects vary between medications. Some adverse effects of antipsychotics can be identified and managed in order that patients can continue therapy while minimizing side effects. To this effect, patients taking antipsychotic medications should be monitored regularly for adverse effects and managed accordingly [1, 10, 16]. The aim of this study was to assess the prevalence and management practice of side effects of first generation antipsychotics among schizophrenic patients in an Ethiopian mental health hospital.

## Materials and methods

We performed a cross sectional study among patients diagnosed with schizophrenia at Amanuel Mental Specialized Hospital in Addis Ababa from March 21 to June 21, 2017. The hospital was established in 1930s and it is the only specialty mental health hospital in the country with a total of 280 beds. Ambulatory Schizophrenic patients visit the psychiatric clinic for follow up every month.

Institutional review board of Addis Ababa University approved the study. Letter of permission to access data was obtained from the clinical director of Amanuel Mental Specialized Hospital. We obtained written informed consent from each participant prior to data collection. The participants were notified of their right to withdraw from the interview at any time of the interview. We assured the participants for confidentiality of their information and the privacy of the respondents was maintained.

### Sample size and sampling method

The sample size for this study was determined by using single population proportion formula [17] with a 95% confidence level and 50% estimated prevalence of first-generation antipsychotics induced side effects and application of correctional formula. Through calculation and 10% compensation for non-response rate, 356 participants were included in the study. The study samples were selected using a systematic sampling technique using a sampling fraction. The sampling fraction (k) was calculated by dividing the total number of schizophrenic patients (2012) having follow up at the hospital by the total sample size (356). Through calculation, every sixth patient was eligible for the study and was interviewed. Out of 356 participants, 300 had complete data and were studied. To maintain the quality of the data, we trained data collectors; English version of the questionnaire was translated to local language and back translated to English. We also pretested the data collection tool.

The diagnosis of schizophrenia was taken from patients’ medical records as confirmed by a treating physician. Participants included in the study were: patients 18 years and above, patients on first generation antipsychotic medication(s) treatment for at least three months and visited the psychiatry clinic during the study period, and patients who were willing to participate. We excluded patients who had acute attack of the illness and were not able to provide reliable information, and those who were on second generation antipsychotics only.

Data on socio-demographic characteristics of the participants, side effects of antipsychotics and adverse drug reactions were collected using a structured questionnaire. Data abstraction format was used to collect data on duration of illness, co-morbid illnesses and medications. Data were collected by trained pharmacists. We used Glasgow Antipsychotic Side Effect Scale (GASS) to assess and compare the side effect burdens of first generation antipsychotics (FGAs). GASS [18] is a patient self-reported measurement scale consisting of twenty two questions that cover a wide range of antipsychotic medications related side effects. Based on GASS score, side effects were categorized as absent or mild side effects if the overall total GASS score was 0–12; moderate side effects if the overall total GASS score was 13–26 and severe side effects if the overall total GASS score was greater than 26.

Naranjo Adverse Drug Reaction Probability Scale (NADRPS) was used to estimate the probability of adverse drug reaction caused by the FGAs [19]. Naranjo Adverse Drug Reaction Probability Scale (NADRPS) involves 10 ‘yes’, ‘no’ or ‘unknown or non-applicable’ questions. The adverse drug reactions were assigned to a probability category on the basis of the total score as ‘definite’ = ≥9, ‘likely’ = 5–8, ‘possible’ = 1–4, ‘unlikely’ < =0.

The main outcome of this study was prevalence of first generation antipsychotic medication(s) related side effects and their management practice.

### Statistical analysis

We analyzed the data using SPSS Version 21.0 (Chicago, SPSS Inc.). We used Chi-square tests to see the association between categorical variables and antipsychotic medications side effects.

### Operational definitions and Definition of terms

**Extrapyramidal Side effects:** are drug-induced movement disorders that include acute and tardive symptoms. These symptoms include dystonia (continuous spasms and muscle contractions), akathisia (motor restlessness), Parkinsonism (characteristic symptoms such as rigidity), bradykinesia (slowness of movement), and tremor, and tardive dyskinesia (irregular, jerky movements [20].

**Definite reaction:** Naranjo adverse drug reaction (ADR) Probability Scale score ≥ 9 [19].

**Probable reaction:** Naranjo adverse drug reaction (ADR) Probability Scale score 5–8 [19].

**Possible reaction:** Naranjo adverse drug reaction (ADR) Probability Scale score 1–4 [19].

**Doubtful reaction:** Naranjo adverse drug reaction (ADR) Probability Scale score ≤ 0 [19].

**Side effect:** Side effect is an undesired effect that occurs when the medication is administered regardless of the dose [20].

**Adverse drug reaction:** is a noxious and unintended reaction that occurs at doses normally used in man for the prophylaxis, diagnosis or therapy of disease, or for modification of physiological function [20].

Sub-therapeutic dose: dose of first generation antipsychotics that is below what is used for treating the disease or producing an optimal therapeutic effect.

## Results

### Baseline characteristics of participants

Out of a total sample size of 356 participants, 300 of them had complete data and were included in the study. The mean age of the study participants was 33.71 ± 10.2 years (range from 18 to 67 years). The majority of the participants were males 195(65.0%). More than half of the participants 166(55.3%) were urban residents. Nearly one-third, 105(35%), completed primary education. One hundred fourteen participants (38.0%) did not have job. Sixty one (20.3%) participants were cigarette smokers (Table 1).

**Table 1:** Socio-demographic characteristics of patients with schizophrenia on follow-up at Amanuel Specialized Mental Hospital

Variable category		Frequency (%)
Age in years	≤35	146 (48.7)
	> 35	154 (51.3)
Sex	Male	195 (65.0)
	Female	105 (35.0)
Residence	Urban	166 (55.3)
	Rural	134 (44.7)
Religion	Orthodox Christian	179 (59.7)
	Muslim	113 (37.7)
	Others	8 (2.7)
Ethnicity	Oromo	89 (29.7)
	Amhara	105 (35.0)
	Gurage	69 (23.0)
	Others	37 (12.3)
Level of education	Primary education	171 (57.0)
	Secondary education	100 (33.3)
	college and above	29 (9.7)
Marital status	Single	150 (50.0)
	Married	111 (37.0)
	Widowed	11 (3.7)
	Divorced	28 (9.3)
Occupation	Jobless	114 (38.0)
	Government employee	28 (9.3)
	Private business	83 (27.7)
	Farmer	45 (15.0)
	Others	30 (10.0)
Substance use	Yes	74 (24.7)
	no	226 (75.3)
Smoke cigarettes	Yes	61 (20.3)
	No	239 (79.9)
Chew khat	Yes	60 (20.0)
	No	240 (80.0)
Drink alcohol	Yes	50 (16.7)
	No	250 (83.3)

As shown in Table 2, the mean duration of schizophrenia was 3.4 ± 1.2 year ranging from six months to ten years. One hundred six (35.3%) participants had schizophrenia for ≥5 years. Thirty two (10.7%) of the participants had comorbid illnesses. The most common comorbidity was major depressive disorder (MDD); 22 (68.8%).

**Table 2:** Clinical characteristics of participants with schizophrenia on follow-up at Amanuel Specialized Mental Hospital

Variable category		Frequency (%)
Duration of illness	< 1 year	17 (5.7)
	1-4 years	177 (59.0)
	≥5 years	106 (35.3)
Comorbidity present	Yes	32 (10.7)
	No	268 (89.3)
Type of comorbidity	MDD	22 (68.8)
	Epilepsy	5 (15.6)
	Others	6 (18.75)

*MDD* Major Depressive Disorder

*Others* include Diabetes mellitus, retroviral infection and hypertension

### Medication regimens and side effect profile of first generation antipsychotics

The most commonly prescribed first generation antipsychotics were chlorpromazine; 114(38.0%) and haloperidol; 53(17.7). The majority of participants, 208(69.3%), were on mono-therapy. Chlorpromazine and Fluphenazine decanoate combination was the most commonly used combination regimen; 55(18.3). The majority of patients, 169(56.3%), were taking < 300 mg Chlorpromazine equivalent dose which is below standard recommendations. One hundred fourteen (38.0%) of patients received therapeutic (300–600 mg) chlorpromazine equivalent dose. The remaining 17 (5.7%) patients were taking supratherapeutic (600–1000 mg) chlorpromazine equivalent dose of FGAs. Two hundred sixty five (88.3%) participants had adequate remission of illness after initiation of first generation antipsychotics (Table 3).

**Table 3:** Type of first generation antipsychotic medications prescribed to treat schizophrenia at Amanuel Specialized Mental Hospital

Variable category		Frequency(%)
Types of antipsychotics	Chlorpromazine	114 (38.0)
	Fluphenazine decanoate	32 (10.7)
	Haloperidol	53 (17.7)
	Thioridazine	4 (1.3)
	Trifluoperazine	4 (1.3)
	Chlorpromazine + Fluphenazine decanoate	55 (18.3)
	Haloperidol + Fluphenazine decanoate	15 (5.0)
	Chlorpromazine + haloperidol	23 (7.7)
Number of antipsychotics	Monotherapy	208 (69.3)
	Combination therapy (≥2 antipsychotics)	92 (30.7)
Chlorpromazine equivalent daily dose of antipsychotics in mg	< 300 (sub-therapeutic)	169 (56.3)
	300–600 (therapeutic)	114 (38.0)
	600–1000 (supra-therapeutic)	17 (5.7)
Response to antipsychotics	Adequate response (remission of illness)	265 (88.3)
	Poor response with recurrent illness	35 (35.0)

As shown in Table 4, most of the participants, 293(97.7%), developed first generation antipsychotic medications related side effects. A total of 821 FGAs related side effects were detected. Eighty four (28.0%) of the participants who developed side effects did not get treatment. One hundred sixty three (54.3%) of the participants who developed antipsychotic medication related side effects were treated with Trihexyphenidyl for the side effects. Dose reduction of antipsychotics was done for, 51(17.0%), of the participants. Nearly two-third (65.3%) of the participants responded to side effect management intervention. The majority of side effects were not managed according to American Psychiatric Association guideline; 178 (82.4%). According to Glasgow antipsychotic side effects rating scale, the majority of participants 165(55.0%), developed mild side effects. Severe side effects were noted in 8 participants.

**Table 4:** Side effects of first generation antipsychotics and management practices at Amanuel Specialized Mental Hospital

Variable category		Frequency(%)
Side effect developed	Yes	293 (97.7)
	No	7 (2.3)
Management of side effects	No side effect management	84 (28.0)
	Dose reduction of antipsychotics	51 (17.0)
	Trihexyphenidyl given	163 (54.3)
	Other interventions	2 (0.7)
Response to side effect management	Responded	20 (6.7)
	No response	196 (65.3)
	Unknown response	84 (28.0)
Side effect managed in accordance with APA guideline	Yes	38 (17.6)
	No	178 (82.4)
Severity of side effects based on GASS	Mild side effects	165 (55.0)
	Moderate side effects	127 (42.3)
	Severe side effects	8 (2.7)
Type of side effect	Cardiovascular	169 (56.3)
	Sedation and CNS	149 (49.6)
	Extrapyramidal	114 (38.0)
	Gastrointestinal	95 (31.7)
	Anti-cholinergic	73 (24.3)
	Diabetes mellitus	73 (24.3)
	Genitourinary	46 (15.3)
	Weight gain	61 (20.3)
	Hyperprolactinemia	41 (13.7)

*APA* American Psychiatric Association

The most common types of FGAs related side effects were cardiovascular side effects 169(56.3%); sedation and CNS side effects149 (49.6%); and extrapyramidal side effects114(38.0%).

### Adverse drug reactions with first generation antipsychotics

According to Naranjo adverse drug reaction (ADR) Probability Scale, 67(22.3%) patients developed a definite first generation antipsychotic medications related adverse drug reaction. Probable adverse drug reaction was noted in 145(48.3%) participants. Seventy five participants (25.0%) developed possible adverse drug reaction. A total of 287 ADRs were recorded out of which 165(57.5%) were dizziness and irregularity in heart beats. Sedation and EPS accounted for 87(30.3%) of ADRs. Other less common ADRs were drooling, dry mouth, sexual dysfunction, and menstrual abnormality. Chlorpromazine was highly linked with sedation and irregularity of heart beats 117(71%). Fluphenazine and haloperidol were the causes for 32(55.2%) and 31(53.4%) extrapyramidal side effects respectively (Table 5).

**Table 5:** The probability of first generation antipsychotics induced adverse drug reactions in patients with schizophrenia at Amanuel Specialized Mental Hospital

Category of ADR; n(%)	Type of ADR (Frequency)	FDAs associated with the ADR
Definite reaction 67 (22.3%)	Dizziness (28)	Chlorpromazine
	Irregularity in heart beat (11)	Chlorpromazine
	Sedation (14)	Chlorpromazine
	EPS (14)	Fluphenazine depot (11) + Haloperidol (8)
Probable reaction 145 (48.3)	EPS (44)	Haloperidol(23) + fluphenazine depot(21)
	Sedation (15)	Chlorpromazine(11) + thioridazine(4)
	Constipation (9)	Fluphenazine depot
	Dizziness (46)	Chlorpromazine(23) + Haloperidol(23)
	Sexual dysfunction (3)	Chlorpromazine(2) + Haloperidol(1)
	Irregularity in heart beat (37)	Chlorpromazine(20) + fluphenazine depot(17)
Possible reaction 75 (25.0)	Irregularity in heart beat (31)	Chlorpromazine (23) + Haloperidol (8)
	Dizziness (12)	Chlorpromazine
	Dry mouth (10)	Chlorpromazine
	Insomnia (9)	Haloperidol
	Menstrual abnormality (5)	Fluphenazine depot
	Drooling (11)	Fluphenazine depot

### Factors associated with the occurrence of side effect of first generation antipsychotics

On chi-square analysis (Table 6), there is a significant association between occurrence of side effects of FGAs and duration of illness (*P* = 0.04). Age, gender, substance use, total daily dose of antipsychotics, presence of co-morbid illness and number of antipsychotics are not significantly associated with the occurrence of side effects.

**Table 6:** Factors associated with the occurrence of first generation antipsychotics related side effects at Amanuel Specialized Mental Hospital

Variable category		Side effect developed		
		Yes	No	X^2^ (P)
		n (%)	n (%)	
Age category in years	≤35	144 (49.1)	2 (28.6)	2.1 (0.72)
	> 35	149 (51.9)	5 (71.4)	
Gender	Male	189 (64.5)	6 (85.7)	1.35 (0.24)
	Female	104 (35.5)	1 (14.3)	
Current substance use	Yes	74 (25.3)	0 (0.0)	2.35 (0.13)
	No	219 (74.7)	7 (100)	
Total daily chlorpromazine equivalent dose of FGAs in mg	< 300	165 (56.3)	4 (57.1)	0.45 (0.8)
	300–600	111 (37.9)	3 (42.9)	
	600–1000	17 (5.8)	0 (0.0)	
Duration of illness in years	< 1	17 (5.8)	0 (0.0)	9.98 (0.04)
	1–5	171 (58.4)	6 (85.7)	
	≥5	105 (35.8)	1 (14.3)	
Presence of comorbidity	Yes	31 (10.6)	1 (14.3)	0.1 (0.75)
	No	262 (89.4)	6 (85.7)	
Number of antipsychotics	1	201 (68.6)	6 (85.7)	0.91 (0.34)
	≥2	92 (31.4)	1 (14.3)	

## Discussion

The majority of the participants (69.3% were on mono-therapy. More than half of the participants (56.3%) were taking low doses of drugs below standard recommendations (< 300 mg Chlorpromazine equivalent dose). Most of the participants (97.7%) developed first generation antipsychotic medications related side effects. A total of 821 FGAs related side effects were detected. Eighty four (28.0%) of the participants who developed side effects did not get treatment. According to Glasgow antipsychotic side effects rating scale, the majority of participants (55.0%) developed mild side effects. Severe side effects were noted in 8 participants. The most common types of FGAs related side effects were cardiovascular side effects (56.3%); sedation and CNS side effects (49.6%); and extrapyramidal side effects (38.0%). There is a significant association between occurrence of side effects of FGAs and duration of illness (*P* = 0.04).

In this study we found that the prevalence of first generation antipsychotics induced side effects was 97.7% out of which 42.3% were moderate side effects according to GASS score. This is a striking finding that the prevalence of antipsychotic medications related side effects was high in our set up. Though there are reports of as high as 86.2% prevalence of antipsychotic medications related side effects [13, 14]. The high magnitude of FGAS associated side effects may be due to the patients’ age and duration of treatment. A report (similar to our finding) showed that relatively younger population groups (mean age 27 years) and those on average duration of treatment 4 years had a significant association with drug-related movement disorders [21]. Combination therapy in 37.7% of the participants and use of substances of abuse in 24.7% of the participants might have contributed to the high prevalence of side effects. Evidences show that illness duration and polypharmacy are significantly associated with the occurrence of side effects [22, 23]. Margaret et al. [9] reported that extrapyramidal side effects with first generation antipsychotics increase three to five times as age increases. In addition, the majority (57%) of the participants in our study attended only primary education. This low level of education might have affected the rate and specification of side effects report. For example, one finding from Ethiopia [24] depicted that chat use, alcohol consumption, and high dose of chlorpromazine (> 400 mg/day) were associated with the occurrence of antipsychotic medications induced movement disorders. Severe psychotic relapse may also be precipitated by illicit Substances that is resistant to medical management which in turn may need high doses of multiple antipsychotic medications to manage it finally putting the patient at risk of developing medication related adverse events [25].

The high level of side effect might significantly affect patient antipsychotic medication adherence and control of schizophrenia. Evidences also showed a strong relation between medication side effects and non-adherence which in turn affect treatment outcome [13, 22, 26, 27]. For example, movement disorders associated with antipsychotics are often associated with disability and distress and may result in disturbances in behavior, medication non-adherence, and exacerbation of schizophrenia and some may be misinterpreted as psychotic symptoms [28].

The most common reported side effects in our study were cardiovascular (56.3%), sedation and CNS (49.6%) and extrapyramidal (38.0%). This finding is against previous findings that reported high prevalence of cognitive-related and extrapyramidal side effects [6, 13, 14, 27]. Evidences [10, 29] also show that extrapyramidal side effects and sedation are the most common side effects associated with first generation antipsychotics.

Reports of Margaret et al. [9] revealed that the prevalence of extrapyramidal side effects was related to higher mean daily and cumulative antipsychotic doses. However, in our set up, though 56.3% of the participants were taking low dose of antipsychotics below standard recommendations, the large proportion of patients developed side effects. This may be due to differences in baseline characteristics of participants and assessment of side effect between ours and other study.

The level of cardiovascular side effects reported in this study is unusually high. Though antipsychotic induced cardiovascular adverse effects has been underreported, some FGAs are less common to cause cardiovascular side effects as compared to atypical ones [30, 31].Studies [32] revealed that the use of Thioridazine is associated with higher risk of cardiovascular adverse effects especially at high doses, but in this study, only 4 patients were on Thioridazine treatment. The high rate of cardiovascular adverse events may be due to poor cardiovascular side effects assessment practices, and failure to confirm the reported side effect with appropriate diagnostic modalities. Moreover, increased age (older than 35 years) of participants in our study might have contributed to FGAs induced side effects.

In our study weight gain and anticholinergic side effects were reported to be 24.3 and 20.3% respectively. Findings from United States [14] and Japan [22] reported a higher prevalence of weight gain ranging from 39 to 42%. The Japanese study [22] also noted higher prevalence of anticholinergic side effects than ours. The lower rate of weight gain in this study may be because our patients are treated with only FGAS which are associated with lower risk of weight gain as compared to newer agents [33] where as in the studies stated newer agents were used in some study participants either as monotherapy or add on therapy. Hashimoto et al. [22] also reported that anticholinergic side effects are significantly high in patients having polypharmacy.

Out of the total participants who developed one or more side effect in our study, 28% of them did not receive management interventions for the side effects. The majority (54.3%) were given Trihexyphenidyl for the management of side effects.

FGAS induced side effects may be managed by different strategies such as lower dose of antipsychotic, administration of anticholinergic medications(including Trihexyphenidyl for extrapyramidal reactions and parkinsonian syndrome), switch to a lower potency antipsychotic medication, and provision of supportive treatment depending on the type of side effect developed [10, 20, 28]. Nevertheless, in this study we noted that Trihexyphenidyl was unjustifiable. It was documented that more than half of the participants were prescribed this drug while the majority of side effects were cardiovascular ones. So there was high use of Trihexyphenidyl for the management of FGA`S induced side effects. The overuse of Trihexyphenidyl for the management of such kind of side effects is also reported in various findings [34, 35]. There is a trend of using anticholinergic medications for schizophrenic patients taking FGAs. In one study, the use of FGAs or use of ≥1 scheduled antipsychotic medications was significantly associated with anticholinergic use in schizophrenic patients [36].

The only factor that is associated with the occurrence of side effects is duration of illness. This evidence is supported by some other findings where duration of schizophrenia had a significant impact on the occurrence of first generation antipsychotic medications related side effects [9, 2].

This study has some limitations. Causal association between antipsychotic medication and side effects was not adequately assessed using laboratory or diagnostic techniques. We only took patient reported side effect. Data on duration of illness was abstracted from patient chart.

## Conclusions

The prevalence of first generation antipsychotics induced side effects was high. The most common first generation antipsychotic medications induced side effects were cardiovascular, sedation and central nervous system, and extrapyramidal side effects respectively. Chlorpromazine was the most common prescribed type of antipsychotic. Duration of schizophrenia was significantly associated with side effects of antipsychotics. Trihexyphenidyl was used by the majority of participants to manage antipsychotic medication(s) induced side effects. Therefore, the hospital should devise strategies to carefully diagnose side effects and manage accordingly to improve patient outcome.

## Abbreviations

ADR: Adverse Drug Reaction
CNS: Central nervous system
EPS: Extrapyramidal side effects
FGAs: First generation antipsychotics
GASS: Glasgow antipsychotic side effect scale
NADRPS: Naranjo adverse drug reaction probability scale
SGAs: Second generation antipsychotics
